# A rare case of relapsed multiple myeloma with aberrant T‐cell antigen expression and skin plasmacytomas

**DOI:** 10.1002/jha2.172

**Published:** 2021-02-07

**Authors:** Catherine S.Y. Lecat, Manuel Rodriguez‐Justo, Dominic Patel, Kwee Yong, Xenofon Papanikolaou

**Affiliations:** ^1^ Research Department of Haematology University College London Cancer Institute London United Kingdom of Great Britain and Northern Ireland; ^2^ Research Department of Pathology University College London Cancer Institute London United Kingdom of Great Britain and Northern Ireland; ^3^ University College London Hospital NHS Foundation Trust London United Kingdom of Great Britain and Northern Ireland

This 60‐year‐old gentleman was first diagnosed with non‐secretory multiple myeloma (MM) with extensive skeletal lytic lesions in March 2017. The t(11;14)(q13;q32) IgH/CCND1 translocation was detected by interphase fluorescence in situ hybridisation (FISH). He was treated with bortezomib, thalidomide and dexamethasone, followed by autologous stem cell transplantation (ASCT). He relapsed at 22 months post‐ASCT with pancytopenia and was commenced on daratumumab, bortezomib and dexamethasone (DVd) in December 2019 with improvement in his blood counts. After three cycles, his treatment was changed to oral cyclophosphamide, lenalidomide and dexamethasone due to COVID‐19 pandemic in order to minimise hospital visits for infusions. Unfortunately, a Positron Emission Tomography‐Computed Tomography (PET‐CT) scan in July 2020 showed disease progression with new extramedullary soft tissue lesions in the right pleural space. In addition, he developed multiple painless skin lesions on his trunk and legs (Figure 1). Skin biopsy showed sheets of pleomorphic cells, immunophenotypically CD138(+), BLIMP1(+), cyclin D1(+), but also positive for CD3, CD4 and CD30 (Figure 2). Restaging bone marrow trephine biopsy contained 10% of atypical cells that lacked plasma cell morphology and no light chain production was seen, but were positive for CD138, BLIMP1 and cyclin D1, consistent with MM. CD3, CD4 and CD30 were also aberrantly expressed; these antigens were not present in his diagnostic marrow sample. Interphase FISH on both bone marrow and skin biopsy demonstrated t(11;14) fusion signals (Figure 2). The patient failed to respond when re‐exposed to DVd, and was subsequently started on a pomalidomide‐based regimen with marked improvement in his skin lesions.

**FIGURE 1:**
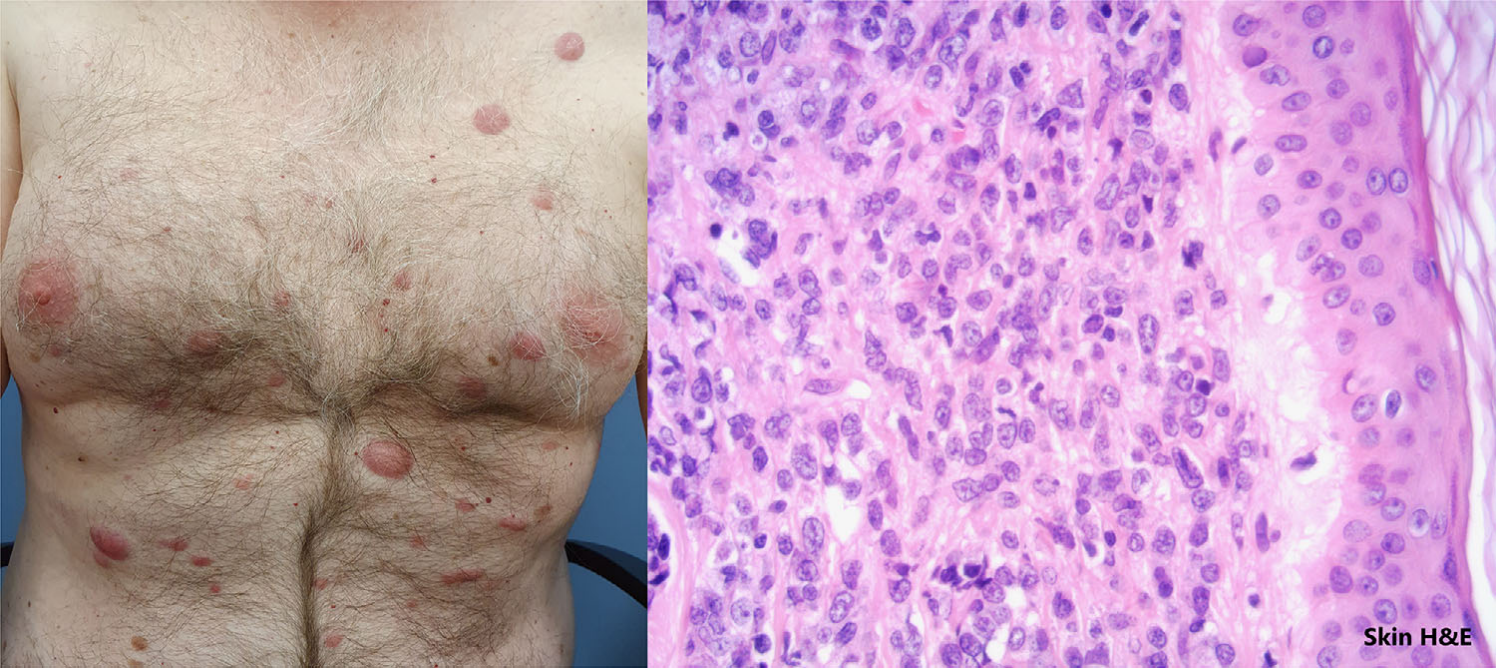
Patient developed erythematous skin lesions on his trunk (left) and legs. On haematoxylin and eosin (H&E) staining of the skin biopsy (right), there were sheets of pleomorphic cells that were similar in morphology and phenotype to the atypical cells found in his bone marrow trephine biopsy at this relapse.

**FIGURE 2:**
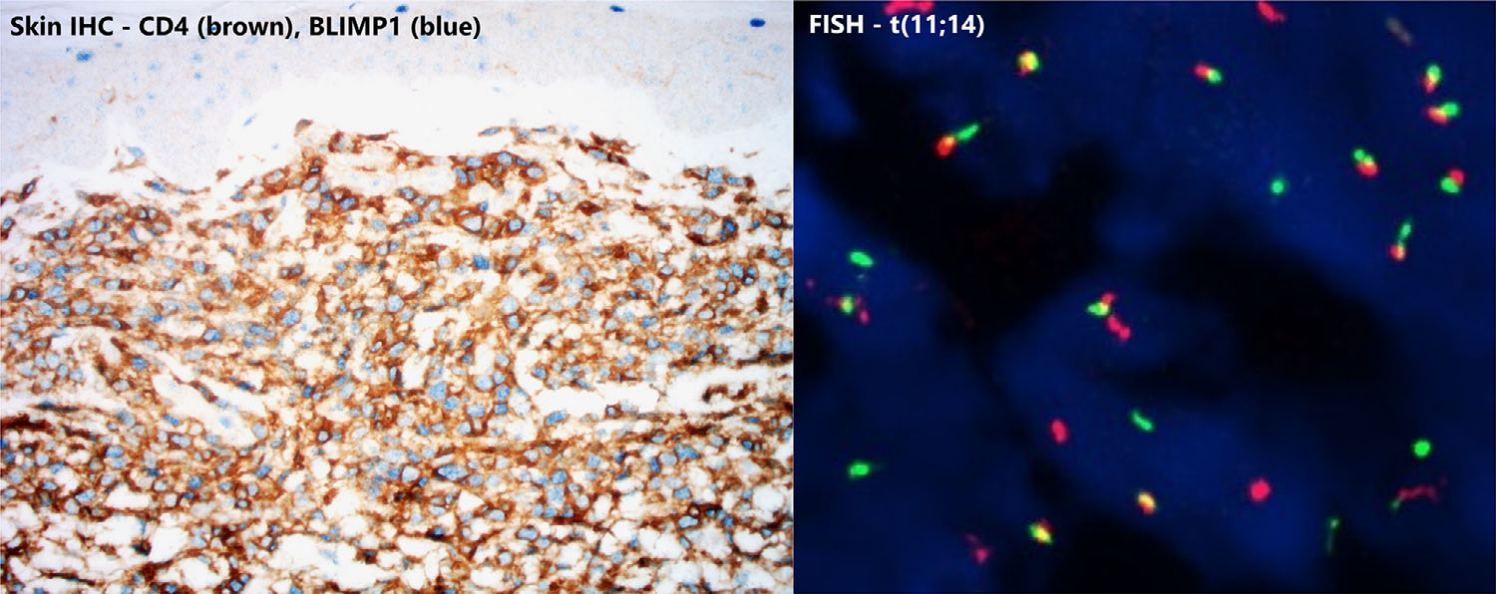
Immunohistochemistry (IHC) staining of the skin biopsy (left) showed that these atypical cells were CD4(+) and BLIMP1(+). They were also CD138(+), cyclin D1(+), CD3(+) and CD30(+). The same pattern was also seen in IHC staining of his bone marrow trephine biopsy. Both the skin and bone marrow biopsies demonstrated t(11;14) fusion signals on interphase FISH (right).

Aberrant expression of T‐cell markers in MM is exceedingly rare, particularly in relapse settings (1‐4). Such expression can pose diagnostic challenges, especially when tumour morphology is ambiguous. The combination of immunohistochemistry (IHC) and FISH for typical MM‐associated aberrations can help distinguish unusual plasma cell neoplasm with T‐cell antigen expression from other differential diagnoses such as plasmablastic lymphoma and NK/T‐cell lymphoma. Skin manifestation in MM is also rare, but may help in the assessment of treatment response in this non‐secretory case, along with repeat imaging.

## AUTHOR CONTRIBUTIONS

Catherine S.Y. Lecat wrote the article; Catherine S.Y. Lecat, Manuel Rodriguez‐Justo and Dominic Patel performed the IHC staining and FISH; Manuel Rodriguez‐Justo, Kwee Yong and Xenofon Papanikolaou read and approved the final version of the manuscript.

## CONFLICT OF INTEREST

The authors declare no conflict of interest.

## Data Availability

The data that support the findings of this study are available from the corresponding author upon reasonable request.
